# NMR in Battery Anode Slurries with a V-Shaped Sensor

**DOI:** 10.3390/s24113353

**Published:** 2024-05-23

**Authors:** Eric Schmid, Louis Kontschak, Hermann Nirschl, Gisela Guthausen

**Affiliations:** 1 Institute of Mechanical Process Engineering and Mechanics, Karlsruhe Institute of Technology, 76131 Karlsruhe, Germany; 2 Chair of Water Chemistry and Water Technology, Engler-Bunte-Institut, Karlsruhe Institute of Technology, 76131 Karlsruhe, Germany

**Keywords:** low-field NMR, inline process monitoring, NMR sensor, battery slurries, flow NMR, relaxation

## Abstract

Inline analytics in industrial processes reduce operating costs and production rejection. Dedicated sensors enable inline process monitoring and control tailored to the application of interest. Nuclear Magnetic Resonance is a well-known analytical technique but needs adapting for low-cost, reliable and robust process monitoring. A V-shaped low-field NMR sensor was developed for inline process monitoring and allows non-destructive and non-invasive measurements of materials, for example in a pipe. In this paper, the industrial application is specifically devoted to the quality control of anode slurries in battery production. The characterization of anode slurries was performed with the sensor to determine chemical composition and detect gas inclusions. Additionally, flow properties play an important role in continuous production processes. Therefore, the in- and outflow effects were investigated with the V-shaped NMR sensor as a basis for the future determination of slurry flow fields.

## 1. Introduction

Nuclear Magnetic Resonance (NMR) is an established and widely used analytical modality with many different applications in various fields of research. The non-invasive, non-destructive method has also been used in industrial quality control [1,2,3]. Low-field NMR is particularly suitable for this purpose as a cost-effective, easy-to-operate, robust and space-saving variant. Despite intensive use in at-line quality control (manual sampling with analytics close to the process), applications of in-line NMR analytics with direct measurements during the process are still rare [4,5,6,7,8].

In addition to applications in electromobility, ongoing energy transition with uneven energy generation from sustainable sources makes larger and more efficient electrochemical storage systems necessary. At the same time, there are also ecological and economical questions regarding the extraction of the necessary raw materials and resources, such as active materials in batteries. Efficient process monitoring and control is required to achieve the best possible performance of the batteries while minimizing the use of resources and costs [9].

The process chain for wet battery electrode production starts with the mixing of the solid components and the solvent in an extruder. The resulting slurry must then be characterized with regard to quality parameters that directly influence the performance of the battery. Examples include the chemical composition of the slurry, gas inclusions, inhomogeneities and rheological properties [10].

NMR sensors with permanent magnets have already been applied in quality control [11] or, for example, in the context of single-sided NMR [12,13,14]. Also, NMR sensors with two parallel disk magnets are known for their use in relaxation and diffusion measurements [15,16]. Inline applications require special hardware geometry for flow-through measurements. The measurement of flow profiles with low-field NMR sensors is shown for example in [17] as well as the quantification of the in- and outflow effects [18].

Based on experiments already carried out ([19] and references therein), the suitability of the V-shaped low-field NMR sensor for investigating the chemical composition of aqueous battery anode slurries and dry mixtures is shown. Also, sensitivity with regard to gas inclusions in the slurry is proven. Flow influences NMR relaxation measurements and provides a basis for rheological measurements with the inline-capable sensor.

## 2. Materials and Methods

The palm-sized V-shaped low-field NMR sensor works at 22.1 MHz and was initially developed with a solenoidal coil in the radiofrequency circuit for offline measurements of liquid samples [19]. Measurements on lubricants in particular have proven the sensors applicability in quality control, and also in an industrial analytical environment. The sensor hardware consists of a V-shaped magnet unit with two NdBFe plate magnets and a radio frequency (rf) probe with a coil and two trimmable capacitors. Control and pulse generation take place via a commercial electronics unit (Bruker ‘the minispec’ ND- and NF-series). The probe contained a solenoidal coil around a PTFE cylinder of 12 mm diameter, in which the sample is positioned. Due to the V-shaped arrangement of the magnet unit, the *B*_0_ field has a magnetic field gradient. Therefore, the NMR measurements were shown to be sensitive to relaxation and diffusion phenomena in the sample.

**Figure 1:**
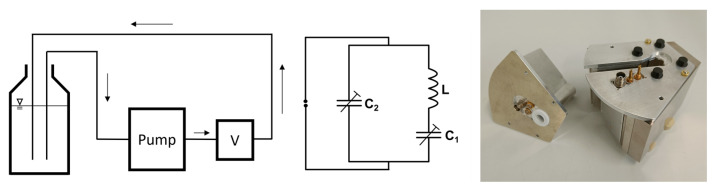
**Left**: Scheme of the experimental setup for flow-through measurements. V symbolizes the V-shaped sensor. **Middle**: The diagram of the electrical resonance circuit. **Right**: Picture of the inline-capable probe that is positioned in the V-shaped magnet unit (in the photo on the right). Please note the slit through which the sensor can be positioned on a pipe with outer diameter *d* < 10 mm. Laying on the left in the photo is a “closed” 12 mm probe with the solenoidal coil.

For an application of the sensor in inline quality control, an “open” probe with a slit and a geometry-adapted coil in the form of a bent figure-8-shaped coil was developed (Figure 1). The sensor can thus be attached to a pipe in a process plant without opening the fluidic system. The use of a dedicated surface coil is necessary for this functionality. The first measurements showed the usability of the sensor in water-based anode slurries for battery production [19].

To show the suitability of flow measurements of anode slurries, an extruded water-based graphite slurry with a solid content of 45%*w*/*w* (96%*w*/*w* graphite, 1%*w*/*w* carbon black (CB), 2%*w*/*w* carboxymethylcellulose (CMC), 1%*w*/*w* styrene-butadiene rubber (SBR)) was used and subsequently diluted to achieve the desired concentrations. To show the principle, the stock slurry was diluted with a water-based Gadovist solution as a contrast agent for faster relaxation processes, with a concentration of 1 mmol/L Gadobutrol (C_18_H_31_GdN_4_O_9_) for the flow measurements.

The sensor was mounted on a vertically positioned tube made of Poly(methyl methacrylate) with an inner diameter of 8 mm and a wall thickness of 0.35 mm for the flow measurements. The tube was connected to a peristaltic pump (Ismatec ISM832C) and the sample reservoir via hoses.

10 mm NMR tubes with sufficiently large filling levels were used for the static measurements, which were performed on water-based anode slurries and also on dry-mixtures to show the feasibility of the sensors’ application in this recently developing field of anode production.

The measurements of the transverse magnetization decays were made via the CPMG pulse sequence (Carr–Purcell–Meiboom–Gill) [20,21] in its low-field version, where whole echo trains can be measured within a single excitation due to the fast *T*_2_* relaxation. Fast measurements are crucial for inline quality control applications with an adequate time resolution.

Depending on the respective question and the sample constitution, different fit models were used to describe and condense the raw data from the CPMG measurements. The monoexponential model was used for pure materials, whereas the biexponential model is optimal for binary mixtures. To describe the magnetization decays of more complex samples, like battery anode slurries, a distribution-based model like the gamma model [22,23] is appropriate. As an additional fit parameter, the distribution width σ provides information about the composition and constitution of the sample [22].

## 3. Results

### 3.1. Determination of the Chemical Composition of Anode Slurries

#### 3.1.1. Measurements on Dry Anode Mixtures

The suitability of the V-shaped sensor for measurements on solid, powdery samples in the form of electrode raw materials was investigated. Transverse relaxation with the CPMG pulse sequence was measured on graphite powder, CMC powder and a mixture of 5%*w*/*w* CMC in graphite powder. The samples were filled in 10 mm NMR tubes with a filling level of 50 mm, high enough to cover the whole sensitive area of the sensor (10 mm).

**Figure 2:**
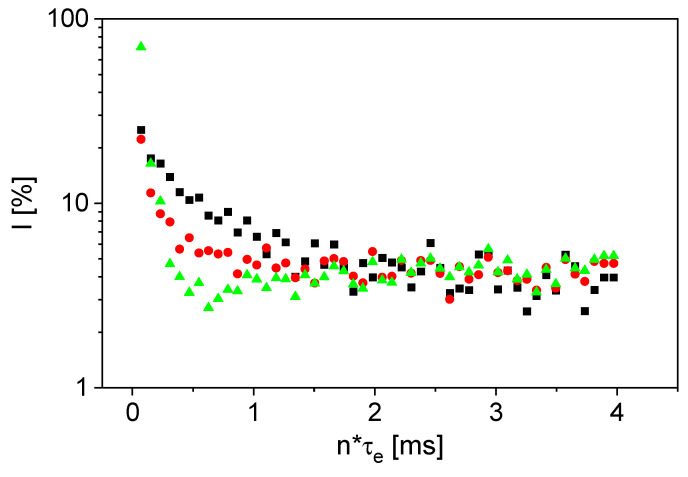
CPMG magnetization decays of graphite powder (■), CMC (▲) and the mixture of graphite and 5%*w/w* CMC (●). Baseline correction and background signal subtraction were applied to show the pure signal decays of the investigated materials.

The magnetization decays (Figure 2) were baseline-corrected to have comparable noise levels. Further, the background signals of the sensor and the sample tube were subtracted, to avoid background signals being interpreted as slurry properties.

The ^1^H signal of the graphite powder is mainly caused by residual moisture. It is observed (Figure 2) that the relaxation of CMC is faster than that of graphite powder. Contributions to the transverse relaxation mainly concern fluctuating dipolar couplings and paramagnetic relaxation, for example due to the iron content in graphite. A detailed analysis of the transverse relaxation would require detailed studies and knowledge about the chemistry, which is out of the scope of this article. The signal of the mixture shows relaxation behavior between the two raw material samples, as expected. The relaxation was quantified via the monoexponential fit model for the raw materials (Table 1). The effective transverse relaxation rate *R*_2,eff_ of CMC is larger by a factor of 4 than *R*_2,eff_ of the graphite powder. The values of *R*_2,eff_ of the raw material samples were then used as fixed parameters in the biexponential fit of the mixture. The amplitudes were then determined by fitting (Table 1). The signal of CMC in the mixture is ten times larger than that of graphite powder, even though the concentration of CMC is only 5%*w*/*w*, reflecting the ^1^H amounts in both substances. The sensor is therefore not only suitable for measurements on liquid samples, but also on powdery mixtures whose composition can be determined.

#### 3.1.2. Measurements on Aqueous Anode Slurries

**Figure 3:**
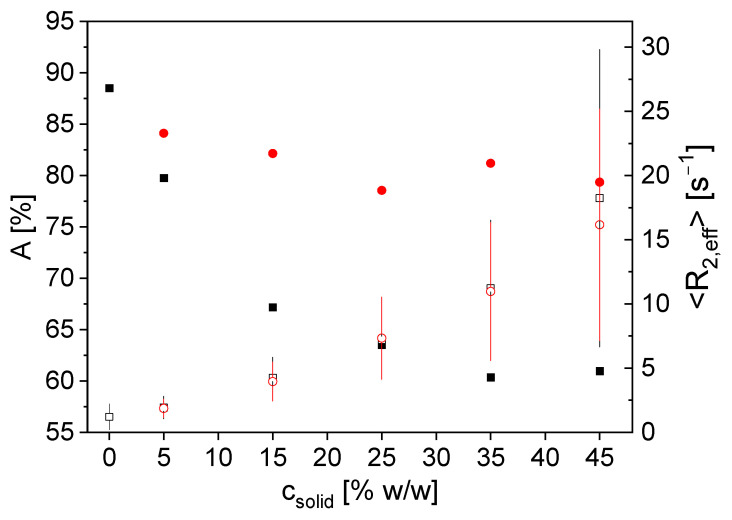
*A* (■, ●) and <*R*_2,eff_> (□, ○) as a function of the solids content in aqueous graphite slurries for measurements with the closed 12 mm probe (**black**) and the open inline-capable probe (**red**). The distribution width σ is shown as pseudo error bars of <*R*_2,eff_>. For both probes, *A* decreases with increasing solids content due to the smaller ^1^H density in the samples; however, this is seen for the closed probe significantly more pronounced than for the open one. <*R*_2,eff_> increases for both probes comparably because of the faster transverse relaxation in the presence of graphite particles and the paramagnetic relaxation enhancement due to the residual iron contamination of graphite.

To prove the sensitivity of the NMR measurements with respect to the aqueous slurry composition, five different samples with different solid contents were measured statically in the closed 12 mm probe, as well as in the open, inline-capable probe. The transverse magnetization decays were measured by the CPMG pulse sequence in combination with a gamma fit model to describe and condense the raw data. The model provides the signal amplitude *A*, the mean effective transverse relaxation rate <*R*_2,eff_> and the distribution width σ of the gamma distribution as fit parameters. The fit parameters are shown as a function of the solids content (Figure 3). The initial slurry with 45%*w*/*w* solids content was diluted with demineralized water. The sample with 0%*w*/*w* solids is pure demineralized water as a reference.

The signal amplitude *A* decays with the solids content for the closed probe. This can be explained by the reduction of ^1^H density due to the larger graphite content in the sensitive area without any ^1^H signal. The non-linearity suggests an influence of graphite particles and paramagnetic impurities beyond the reduction of the ^1^H density. *A* decreases much less for the open, inline-capable probe. This is due to the smaller measurement depth of the bent figure-8-shaped coil [19]. Due to sedimentation in the sample during the measurement, the particles tend to migrate towards the center of the sample tube rather than near the tube’s surface in the used set-up [24]. This leads to a smaller decrease in *A* as a function of the solids content in the sensitive region of the open probe. <*R*_2,eff_> increases with increasing solids content for both probes due to the increasing influence of the iron-containing graphite particles on the transverse magnetization decay. Paramagnetic impurities in the graphite accelerate the relaxation by means of paramagnetic relaxation enhancement PRE. The concentration amounts up to 50 ppm iron in graphite. The distribution width σ of the gamma distribution also increases with increasing solids content. This can also be explained by PRE and less free water with increasing solids content.

The sensor measurements show good sensitivity towards the chemical composition of the slurry. Not only can the reduction in ^1^H density be measured, but also the influence of the solid ingredients on the integral relaxation of the entire sample. Regarding *A*, the results differ for the two probes due to the different coil geometry, which has an impact on sensitive volume, pulse properties and sensitivity. Regarding <*R*_2,eff_>, the measurements are reproduceable and nearly independent of the probe and filling of the sensitive volume.

### 3.2. Inline Detection of Gas Inclusions

**Figure 4:**
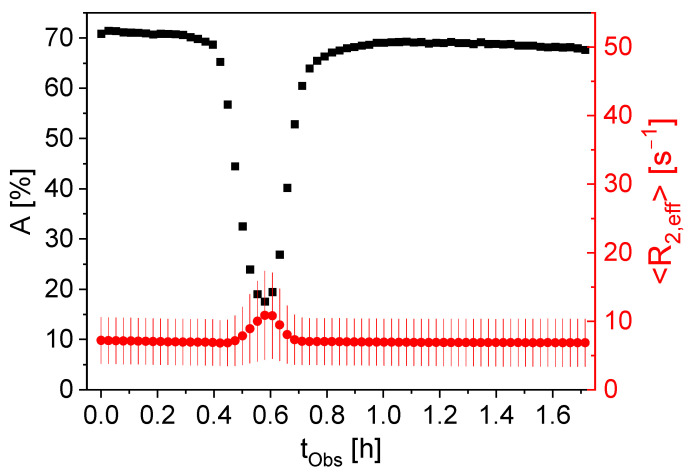
*A* (■) and <*R*_2,eff_> (●) as a function of observation time *t*_Obs_ for a static graphite slurry of 30%*w/w* graphite through which an air bubble moves slowly, driven by gravity. The distribution width σ is shown as red error bars of <*R*_2,eff_>. *A* reflects the presence of the air bubble: *A* is smaller by a factor of 4.7 when the air bubble is in the NMR-sensitive volume due to the then-smaller ^1^H density. <*R*_2,eff_> is larger because of a larger contribution from the sediment (water + graphite) with a significantly faster transverse ^1^H relaxation than pure water to the NMR signal.

The appearance of gas bubbles in electrode slurries directly influences the subsequent processing steps in electrode production and, as a consequence, the quality of the electrode and the performance of the final battery. The inline detection of possible gas inclusions is therefore an important feature of the sensor. To prove the sensitivity of the sensor to gas bubbles, a sample tube with a diameter of 10 mm was positioned horizontally in the sensitive area of the sensor equipped with the closed probe (solenoidal coil). The tube was filled with graphite slurry with a solids content of 30%*w*/*w*. By introducing an air bubble into the slurry and by slightly tilting the sensor and the sample, the bubble was allowed to move slowly through the sensitive area. Transverse relaxation measurements were made at defined time intervals during the experiment. The fit parameters *A*, <*R*_2,eff_> and σ were determined within the gamma fit model to describe and condense the magnetization decays (Figure 4).

When the air bubble is in the sensitive area (section in the sensor where excitation and detection take place), the signal amplitude decreases by a factor of 4.7. This is due to the smaller ^1^H density in the sensitive volume when the air bubble passes. Simultaneously, <*R*_2,eff_> increases by a factor of 1.6. The air bubble displaces water at the top of the tube. As a result, there is a larger contribution to the NMR signal from the sediment, consisting of water and graphite particles, with a faster transverse relaxation than pure water. The relaxation rate distribution width σ also increases when the air bubble passes. The experimental results prove the sensitivity of the measurements on gas inclusions in the sample, whereby all fit parameters are sensitive.

### 3.3. Measurement of Flow Velocity Distributions Exploring the In- and Outflow Effects

**Figure 5:**
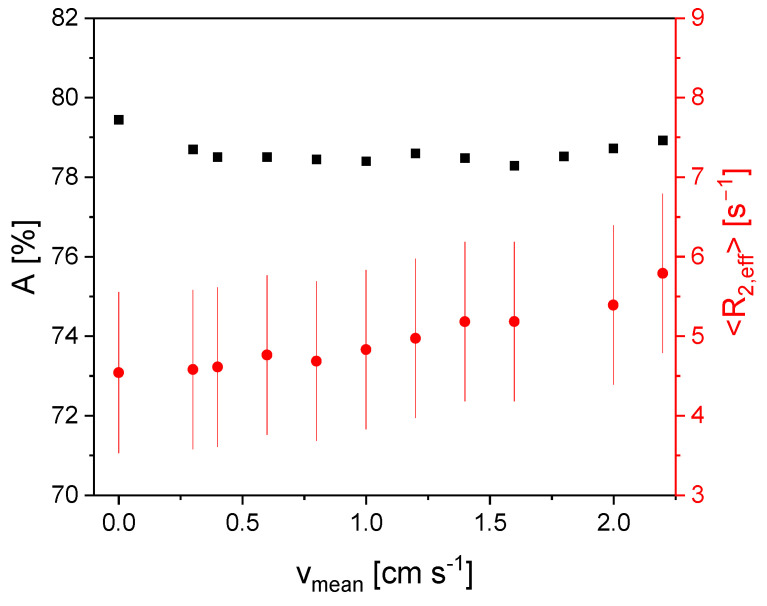
*A* (■) and <*R*_2,eff_> (●) for an aqueous Gadovist solution as a function of the mean flow velocity *v*_mean_. The relaxation distribution width σ is again shown as pseudo error bars of <*R*_2,eff_>. *A* more or less is on a plateau and shows almost no dependence on the flow velocity except for *v*_mean_ = 0. <*R*_2,eff_> linearly depends on *v*_mean_ which is a consequence of the outflow effect.

In- and outflow effects are well known and commonly explored in NMR flow measurements [25]. It is expected that the signal intensity decreases with increasing flow velocity due to the inflow effect. The outflow effect causes the effective transverse relaxation rate to increase with the flow velocity. The expression of the effects should be sensitive to the composition of the sample. Faster longitudinal relaxation leads to a less pronounced inflow effect, whereas faster transverse relaxation reduces the outflow effect. To study how these properties affect the measurements with the V-shaped NMR sensor, CPMG flow measurements were carried out with variable flow velocities of an aqueous Gadovist solution. The fit parameters of the gamma fit model reflect both in- and outflow shown as a function of the mean flow velocity (Figure 5).

The amplitude *A* (Figure 5) shows almost no dependence on the flow velocity except for *v*_mean_ = 0 cm/s. No significant inflow effect is detectable for the Gadovist solution in the investigated velocity range. *T*_1_ is sufficiently small, so the residence time in the *B*_0_ field leads to a complete magnetization of the nuclei. <*R*_2,eff_> increases with increasing flow velocity and shows a linear dependence, which is caused by the outflow effect. This increase shows the sensitivity of the measurements to flow velocity directly. The findings on the Gadovist sample are used as a reference for the more complicated fluids in the following.

**Figure 6:**
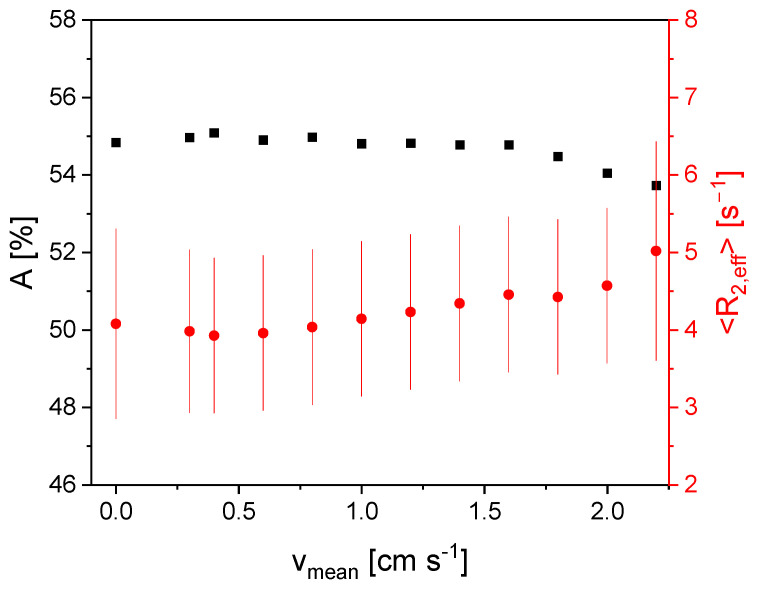
*A* (■) and <*R*_2,eff_> (●) for a graphite slurry with a solids content of 9%*w*/*w* as a function of *v*_mean_. The relaxation distribution width σ is shown as error bars of <*R*_2,eff_>. *A* is on a plateau for *v*_mean_ < 1.5 cm/s. For *v*_mean_ > 1.5 cm/s, *A* decreases, which is a consequence of the inflow effect. <*R*_2,eff_> shows a plateau for smaller velocities and then shows a linear dependence on *v*_mean_.

For the diluted graphite slurry with a solids content of 9%*w*/*w*, *A* is on a plateau for mean flow velocities smaller than 1.5 cm/s (Figure 6). *A* decreases for larger *v*_mean_. This is a consequence of the inflow effect, due to longer longitudinal relaxation times compared to the Gadovist sample. The residence time in the *B*_0_ field depends on the fluids velocity field and leads to an incomplete polarization prior to excitation for the aqueous slurry. Despite the small solids proportion in this fluid, the influence of the inflow effect is evident (Figure 6), while the behavior of the mean effective transverse relaxation rate does not reflect the small solids content and its impact on the flow behavior and will be discussed in the following.

<*R*_2,eff_> is on a plateau for *v*_mean_ < 0.6 cm/s and is slightly smaller than in the Gadovist sample due to the dilution as a consequence of sample preparation (20%*w*/*w* (slurry with 55%*w*/*w* water) + 80%*w*/*w* Gadovist solution). There is a linear dependence of <*R*_2,eff_> for larger velocities due to the outflow effect, which is given by the transverse relaxation. Except for *v*_mean_ = 0 cm/s and *v*_mean_ = 2.4 cm/s, the distribution width of the relaxation rate distribution is constant, and only for the two edge values is it larger.

**Figure 7:**
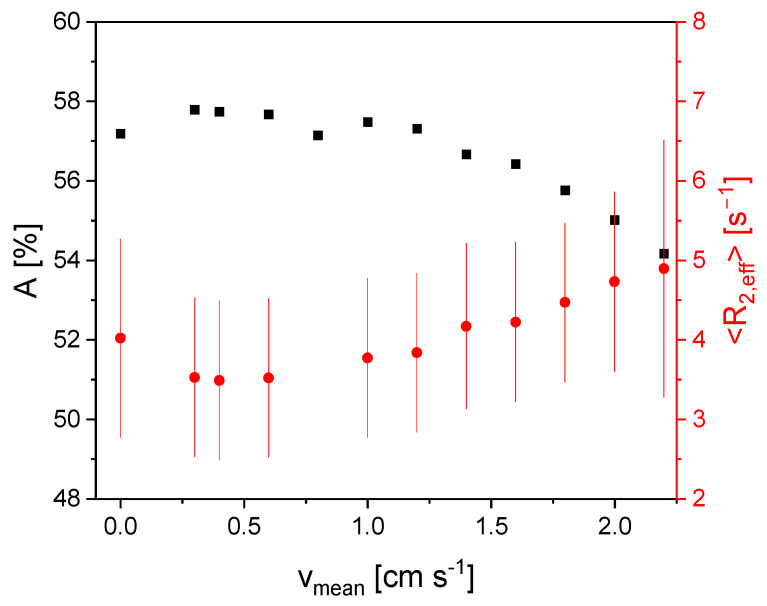
*A* (■) and <*R*_2,eff_> (●) as functions of the mean flow velocity for a graphite slurry with a solids content of 13.5%*w*/*w*. The distribution width σ is shown as error bars of <*R*_2,eff_>. *A* is on a plateau for *v*_mean_ < 1.0 cm/s. The “break” point appears at lower *v*_mean_ because of the larger dilution of Gadovist. <*R*_2,eff_> shows a minimum value for *v*_mean_ = 0.4 cm/s. There is an approximately linear dependence of <*R*_2,eff_> (*v*_mean_) for larger velocities.

In addition, a graphite slurry with a larger solids content of 13.5%*w*/*w* was investigated, and the results differ significantly for the findings in Figure 5 and Figure 6. *A* is on a plateau until *v*_mean_ = 1.0 cm/s (Figure 7). For larger velocities, *A* shows a decrease. The inflow effect is thus already evident at lower velocities because of the smaller longitudinal relaxation rate of the sample with a larger amount of graphite slurry, i.e., showing a slower magnetization build-up compared to the Gadovist sample.

<*R*_2,eff_> shows a minimum for *v*_mean_ = 0.4 cm/s and increases approximately linearly for higher velocities in accordance with the expected combined in- and outflow effects. The slope of <*R*_2,eff_> (*v*_mean_) is larger than for the other samples. This indicates a more pronounced outflow effect due to the slower transverse relaxation of the more concentrated slurry compared to the other samples (Figure 5 and Figure 6). This seems to contradict the static measurements (Figure 3). However, it should be noted that the samples for the static measurements were diluted with water, while the samples for the flow measurements were diluted with Gadovist solution, which explains this fact.

The experiments concerning the in- and outflow effects prove the sensitivity of the V-sensor measurements regarding the flow of graphite slurries even for the bent figure-8-shaped coil with its inherently limited measurement depth. Both the inflow and the outflow effect allow the mean flow velocity of the sample to be determined. Additionally, the two effects are sensitive to the sample composition, so several properties of the sample can be determined at the same time: longitudinal and transverse relaxation as well as solids content and flow. By repositioning the coil and thus the sensitive area in the magnet unit along the flow direction, the pre-polarization length and thus the magnetization time of the flowing sample can be adjusted. In this way, the intensity of the inflow effect and therefore the sensitivity can be adjusted according to the material properties.

## 4. Conclusions

The application of the V-sensor in this paper is focused on the quality control of graphite anode slurries in battery production. In addition to the sensitivities described in [19], the sensor measurements enable (a) the determination of the solids content in the sample, which is an important quality parameter in battery production, (b) the sample’s chemical composition, deduced from transverse relaxation (solids vs. liquids) and (c) the sensitivity of the CPMG measurements towards the flow properties of the sample. In more detail, the possibility of detecting other lower concentrated ingredients, like CMC, in a dry mixture could be proven as well as (d) the ability of the sensor to detect gas inclusions in the slurry, which is desirable information during feed-forward information transfer in a process chain like in the coating of the electrodes. Measurements on anode slurries of different concentrations were made in the flow-through mode, and the impact of in- and outflow effects were investigated as a basis for future measurements aimed at the rheological behavior of the slurries. The investigations carried out show the suitability of the sensor for in-line applications in quality control during the production of battery slurries. Several relevant quality parameters of slurries are available by measuring the relaxation behavior in an inline and non-destructive manner. The determination of flow profiles with the sensor will be the subject of further investigations.

## Figures and Tables

**Table 1:** The monoexponential (raw materials) and biexponential (powder mixture) fit parameters for the solid-state measurements on the respective materials. *A* is the signal amplitude that was determined from the raw data with the respective fit model.

Parameter	Graphite Powder	CMC Powder	Powder Mixture
*A*_Graphite_ [%]	22	-	3
*A*_CMC_ [%]	-	100	29
*R*_2,eff,Graphite_ [ms^−1^]	2.38	-	2.38
*R*_2,eff,CMC_ [ms^−1^]	-	9.71	9.71

## Data Availability

The data are available on request to the authors.
